# Association between weight-adjusted-waist index and the risk of hyperuricemia in adults: a population-based investigation

**DOI:** 10.3389/fendo.2023.1236401

**Published:** 2023-10-11

**Authors:** Yunyi Ding, Zhuohan Xu, Xue Zhou, Yichen Luo, Ruijie Xie, Yayu Li

**Affiliations:** ^1^ Department of Nephrology, Hangzhou TCM Hospital of Zhejiang Chinese Medical University, Hangzhou, China; ^2^ Department of Nephrology, The First Clinical Medical College of Zhejiang Traditional Chinese Medical University, Hangzhou, China; ^3^ School of Mechanical Engineering, Zhejiang University, Hangzhou, China; ^4^ Division of Clinical Epidemiology and Aging Research, University of Heidelberg, Heidelberg, Germany; ^5^ Department of Nephrology, Hangzhou TCM Hospital, Hangzhou, China

**Keywords:** weight-adjusted-waist index, obesity, hyperuricemia, NHANES, cross-sectional study

## Abstract

**Objective:**

This investigation sought to elucidate the potential correlation between a recently characterized adiposity metric, termed the Weight-Adjusted-Waist Index (WWI) and hyperuricemia.

**Methods:**

A cross-sectional design was employed in this study, featuring both hyperuricemic and non-hyperuricemic subjects with complete WWI data, sourced from the National Health and Nutrition Examination Survey (NHANES) spanning 2017 to March 2020. WWI was calculated utilizing the formula which involves the division of waist circumference (WC) by the square root of the body weight. In order to determine the relationship between WWI and hyperuricemia, both univariate and multivariate logistic regression models, appropriately weighted, were employed in the analysis. The linearity of relationships was validated using smooth curve fitting. Additionally, subgroup evaluations and interaction assessments were conducted.

**Results:**

The study sample comprised 7437 subjects, yielding a hyperuricemia prevalence of 18.22%. Stratifying WWI into tertiles, a progressive rise in hyperuricemia prevalence was evident with increasing WWI (Tertile 1: 11.62%, Tertile 2: 17.91%, Tertile 3: 25.13%). The odds ratio (OR) demonstrated that individuals within the highest WWI tertile were significantly more prone to hyperuricemia than those in the lowest tertile (OR = 2.41, 95% CI: 1.88-3.08).

**Conclusion:**

This study provides evidence that an elevated WWI is correlated with an increased risk of hyperuricemia in the adult population of the United States. These results suggest that WWI may serve as a viable anthropometric indicator for predicting hyperuricemia.

## 1 Introduction

Uric acid serves as the terminal product of both endogenous and dietary purine metabolism. Generally speaking, hyperuricemia is characterized by serum uric acid (SUA) concentration exceeding 7.0 mg/dL in men or over 6.0 mg/dL in women. With a burgeoning prevalence worldwide, particularly in developed nations, hyperuricemia is often attributed to the consumption of more dietary-purine-rich foods, more saturated fats, more fructose-enriched beverages, and more alcohol. Presently, approximately one-fifth of the United States population is affected by hyperuricemia (1). Predominantly arising from diminished renal excretion and urate overproduction, notably observed in conditions such as tumor lysis syndrome and metabolic disorders. Hyperuricemia is well-acknowledged for escalating the risk of gout and kidney stones. Furthermore, recent epidemiological inquiries have demonstrated its association with chronic kidney disease, metabolic syndrome, hypertension, insulin resistance, diabetes, and cardiovascular disease progression (2, 3). It has also been observed with various comorbidities including hypertriglyceridemia, hypercholesterolemia, abdominal obesity, and general obesity (4). Consequently, hyperuricemia imposes substantial health burdens on the public health infrastructure.

Obesity, a result of intricate interactions between genetic, metabolic, behavioural, and environmental factors, has emerged as a major health concern over the past century (5). Forecasts suggest that by 2030, nearly half of the adult population will be classified as obese (6). Obesity is linked to numerous health conditions, including hypertension, diabetes, cardiovascular disease, and cancer (7). The contemporary understanding perceives obesity as a complex, heterogeneous chronic disease presenting variably among patients. They need to necessitate individualized, long-term management likely to other complex chronic diseases (8). Therefore, an effective and precise parameter for obesity assessment is of paramount importance.

At present, the body fat indices commonly used in the clinical assessment of obesity have their limitations. The Body Mass Index (BMI) is a commonly employed tool to evaluate and categorize obesity. However, it fails to differentiate between body fat and lean body mass or between central and peripheral fat. Waist circumference (WC) has demonstrated independent associations with increased cardiovascular risk, yet its predictive ability for visceral adipose tissue at an individual level remains limited; Waist-to-hip ratio (WHR) and waist-to-height ratio (WHtR) can more accurately describe abdominal obesity, however, these measures also fall short in distinguishing between subcutaneous and visceral fat (9). The Weight-Adjusted-Waist Index (WWI), a novel obesity metric proposed by Park et al. (10), is derived from WC/weight^1/2^. WWI leverages the benefits of WC, simultaneously diluting its correlation with BMI, thus primarily reflecting central obesity (11). Prior studies have suggested that WWI possesses robust predictive power for cardiometabolic morbidity and mortality (10). Additionally, a Korean study indicated a positive correlation between WWI and fat mass but a negative correlation with muscle mass (12). Therefore, WWI as a new clinical index could potentially enhance the precision of obesity categorization and risk prediction, thus paving the way for more tailored therapeutic interventions and monitoring strategies.

A close relationship exists between obesity and hyperuricemia, with several studies identifying an association between BMI and the risk of hyperuricemia (13–16). However, the association between WWI and hyperuricemia prevalence remains unexplored. Hence, this study aims to examine the correlation between WWI and hyperuricemia, leveraging data from the National Health and Nutrition Examination Survey (NHANES) from 2017 to March 2020.

## 2 Methods

### 2.1 Study population

The data under examination in this study was collected by the National Center for Health Statistics at the Centers for Disease Control and Prevention during the NHANES period from 2017 to March 2020. This cross-sectional study adopted a stratified, multistage probability cluster sampling approach, thereby ensuring a representative sample of the non-institutionalized civilian population in the United States is included in our study (17). The study protocol received approval from the Research Ethics Review Board at the National Center for Health Statistics (NCHS). Prior to participation, all subjects provided informed written consent.

**Figure 1:**
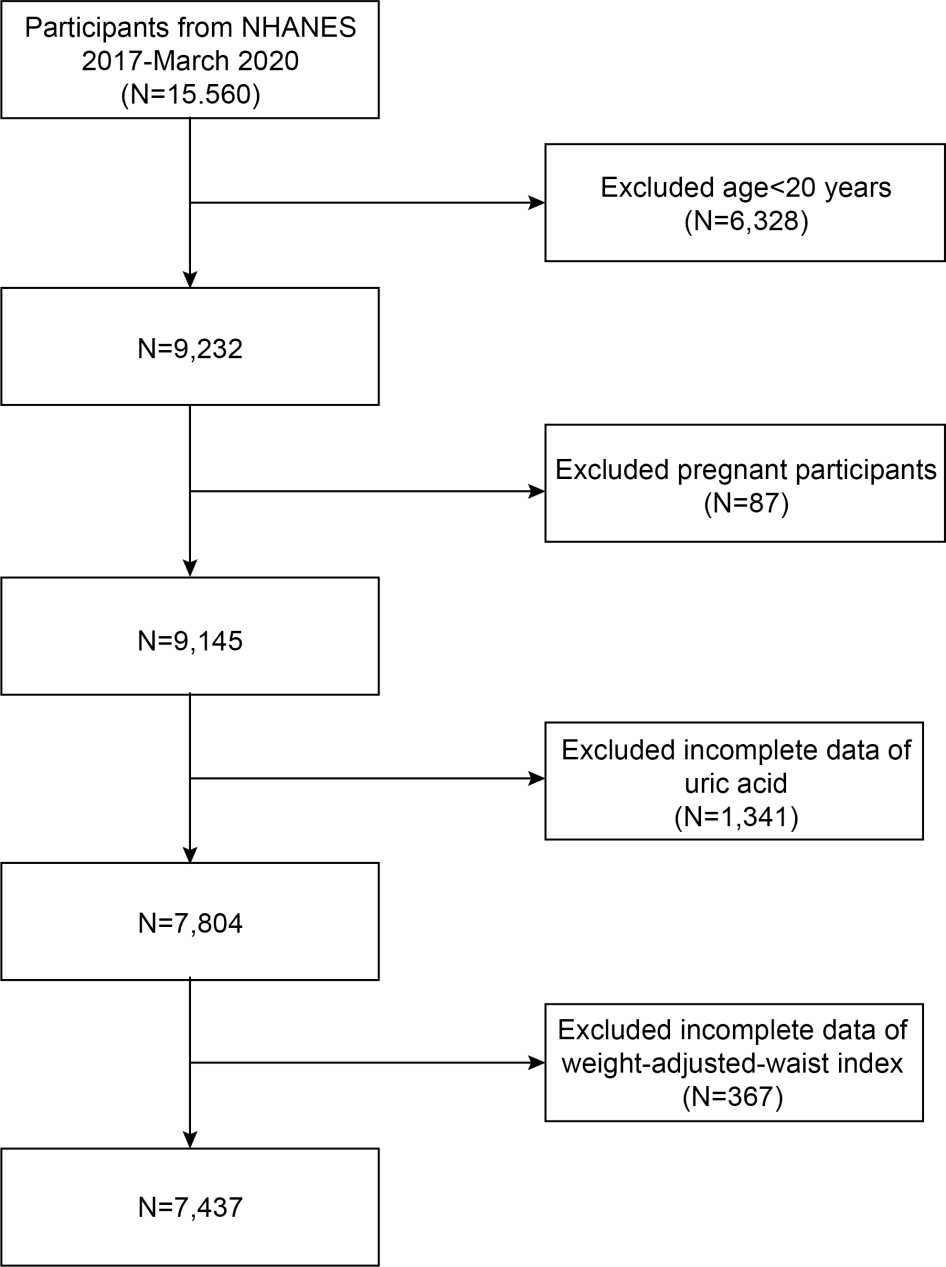
Flow chart of participants selection. NHANES, National Health and Nutrition Examination Survey.

Initially, a total of 15560 subjects were incorporated into the study. However, exclusions were made for those under 20 years of age (N=6328), pregnant individuals (N=87), those without available uric acid information (N=1341) and WWI (total, N=367; WC, N=362; weight, N=5). Subsequently, our final analysis comprised a comprehensive dataset of 7,437 eligible adults. (**Figure 1**).

### 2.2 Study variables

WWI is an anthropometric metric based on WC and body weight, utilized for central obesity assessment. In the NHANES study, trained health technicians accurately measured the WC and body weight. Each participant’s WWI was calculated utilizing the formula which involves the division of WC by the square root of the body weight, rounding the resulting value to two decimal places (WWI = WC/body weight^1/2^, where WC is in cm and body weight is in kg). A higher WWI implies a greater degree of obesity. In this analysis, WWI was utilized as an exposure variable.

Serum uric acid was ascertained using a Roche Cobas 6000 (c501 module) between the years 2017 to March 2020. Hyperuricemia was classified according to SUA levels, adopting thresholds of greater than 7 mg/dL for men and over 6 mg/dL for women (18). This served as the outcome variable in our investigation.

Additionally, a series of covariates were considered in the analysis, including age, gender, race, education, income-to-poverty ratio, smoking status, and alcohol consumption. Anthropometric and laboratory covariates were also incorporated, such as fasting blood glucose (mg/dL), glycated hemoglobin (%), white blood cell count (1000 cells/µL), platelet count (1000 cells/µL), hemoglobin (g/dL), total bilirubin (mg/dL), serum creatinine (mg/dL), total cholesterol (mg/dL), triglycerides (mg/dL), high-density lipoprotein cholesterol (mg/dL), and low-density lipoprotein cholesterol (mg/dL). Hypertension was identified based on any of the following criteria: an existing prescription for antihypertensive medication, a confirmed medical diagnosis of hypertension, or a recorded systolic blood pressure of 140 mmHg or higher, or diastolic blood pressure of 90 mmHg or higher, documented on three consecutive measurements (19). It is essential to highlight that lipid profile measurements were performed exclusively on participants who adhered to a fasting period of no less than 8.5 hours and no more than 24 hours, thereby ensuring the accuracy of these assessments. All data pertaining to these variables are publicly accessible at https://www.cdc.gov/nchs/nhanes/ (accessed June 1, 2023).

### 2.3 Statistical analysis

The statistical analyses in this study were conducted using R software (version 4.2) and EmpowerStats (version 4.1). Participant demographic characteristics, categorized by WWI tertiles, were analyzed using Chi-square tests for categorical variables and t-tests for continuous variables. The association between WWI and hyperuricemia was evaluated using multivariate logistic regression analyses. To examine potential nonlinear relationships between WWI and hyperuricemia, we utilized a weighted generalized additive model along with smooth curve fitting. Mediation analysis was performed using the parallel mediation model, with individual indicators serving as mediators. Further stratified analyses and interaction tests were implemented to ascertain the association between WWI and hyperuricemia across different population segments. Statistical significance was set at a two-sided P-value less than 0.05.

## 3 Results

### 3.1 Baseline characteristics

The analysis included a total of 7437 participants, averaging an age of 50.71 ± 17.35 years. The gender distribution was relatively balanced with 48.81% males and 51.19% females. The participants were divided into three tertiles based on WWI values, namely 8.51-10.77 (Tertile 1), 10.78-11.50 (Tertile 2), and 11.50-14.34 (Tertile 3). This study incorporated 3630 male and 3807 female participants. The overall prevalence of hyperuricemia was 18.22% (weighted proportion), which was observed to escalate with increasing WWI tertile (Tertile 1: 11.62%; Tertile 2: 17.91%; Tertile 3: 25.13%; P<0.001). Significant differences were identified across WWI tertiles with respect to age, gender, race, education, income-to-poverty ratio, smoking habits, drinking habits, hypertension, and serum creatinine levels (all P < 0.05). The findings indicate that participants in the highest tertile of WWI were more likely to be female, non-Hispanic white, have a lower level of education, and be diagnosed with hypertension. Additionally, it was observed that as age and serum creatinine levels increased, so did the WWI (**Table 1**).

**Table 1:** Baseline characteristics of the study population according to weight-adjusted-waist index tertiles.

Weight-adjusted-waist index	Tertile 1	Tertile 2	Tertile 1	*P* for trend
	(8.51-10.77)	(10.78-11.50)	(11.50-14.34)	
	N=2479	N=2479	N=2479	
**Age(years)**	40.94 ± 15.04	51.95 ± 16.15	59.26 ± 15.69	<0.001
**Sex, n (%)**				<0.001
Male	1431 (57.72%)	1293 (52.16%)	906 (36.55%)	
Female	1048 (42.28%)	1186 (47.84%)	1573 (63.45%)	
**Race/ethnicity, n (%)**				<0.001
Mexican American	240 (9.68%)	328 (13.23%)	325 (13.11%)	
Other Hispanic	259 (10.45%)	260 (10.49%)	251 (10.13%)	
Non-Hispanic White	774 (31.22%)	893 (36.02%)	980 (39.53%)	
Non-Hispanic Black	640 (25.82%)	613 (24.73%)	629 (25.37%)	
Other Races	566 (22.83%)	385 (15.53%)	294 (11.86%)	
**Education level, n (%)**				<0.001
Less than high school	293 (11.82%)	469 (18.94%)	589 (23.81%)	
High school or GED	578 (23.33%)	567 (22.90%)	651 (26.31%)	
Above high school	1607 (64.85%)	1440 (58.16%)	1234 (49.88%)	
**Income to poverty ratio, n (%)**				<0.001
<1.31	560 (26.01%)	567 (26.08%)	664 (31.13%)	
1.31–3.50	772 (35.86%)	835 (38.41%)	936 (43.88%)	
>3.50	821 (38.13%)	772 (35.51%)	533 (24.99%)	
**Smoking behavior, n (%)**				<0.001
Never	1 (0.11%)	1 (0.09%)	2 (0.19%)	
Former	379 (41.42%)	635 (59.35%)	671 (64.27%)	
Now	535 (58.47%)	434 (40.56%)	371 (35.54%)	
**Drinking behavior, n (%)**				<0.001
No	162 (6.82%)	198 (8.33%)	266 (11.30%)	
Yes	2215 (93.18%)	2178 (91.67%)	2087 (88.70%)	
**Hypertension, n (%)**				<0.001
No	2079 (83.86%)	1774 (71.56%)	1616 (65.19%)	
Yes	400 (16.14%)	705 (28.44%)	863 (34.81%)	
**Fast glucose (mg/dL)**	110.15 ± 35.18	109.87 ± 34.23	112.65 ± 39.05	0.296
**Glycohemoglobin (%)**	5.76 ± 1.18	5.74 ± 1.02	5.80 ± 1.07	0.067
**White blood cell (1000 cells/μL)**	7.30 ± 2.24	7.41 ± 9.12	7.28 ± 2.28	0.401
**Hemoglobin(g/dL)**	13.70 ± 1.56	13.69 ± 1.53	13.76 ± 1.50	0.641
**Platelets (1000 cells/μL)**	264.98 ± 73.77	262.76 ± 75.10	264.72 ± 73.89	0.53
**Serum total bilirubin (mg/dL)**	0.45 ± 0.27	0.46 ± 0.29	0.45 ± 0.27	0.384
**Serum creatinine (mg/dL)**	0.86 ± 0.42	0.89 ± 0.55	0.89 ± 0.51	0.038
**Total cholesterol (mg/dL)**	181.81 ± 44.29	179.32 ± 40.65	183.47 ± 43.18	0.698
**Triglyceride (mg/dL)**	99.27 ± 66.43	97.93 ± 68.55	103.54 ± 60.65	0.22
**High-density lipoprotein cholesterol (mg/dL)**	55.32 ± 18.67	52.66 ± 13.88	52.29 ± 15.15	0.33
**Low-density lipoprotein cholesterol (mg/dL)**	105.61 ± 37.80	107.03 ± 35.18	109.87 ± 36.74	0.414
**Hyperuricemia, n (%)**				<0.001
No	2191 (88.38%)	2035 (82.09%)	1856 (74.87%)	
Yes	288 (11.62%)	444 (17.91%)	623 (25.13%)	

### 3.2 Association between WWI and hyperuricemia

**Figure 2:**
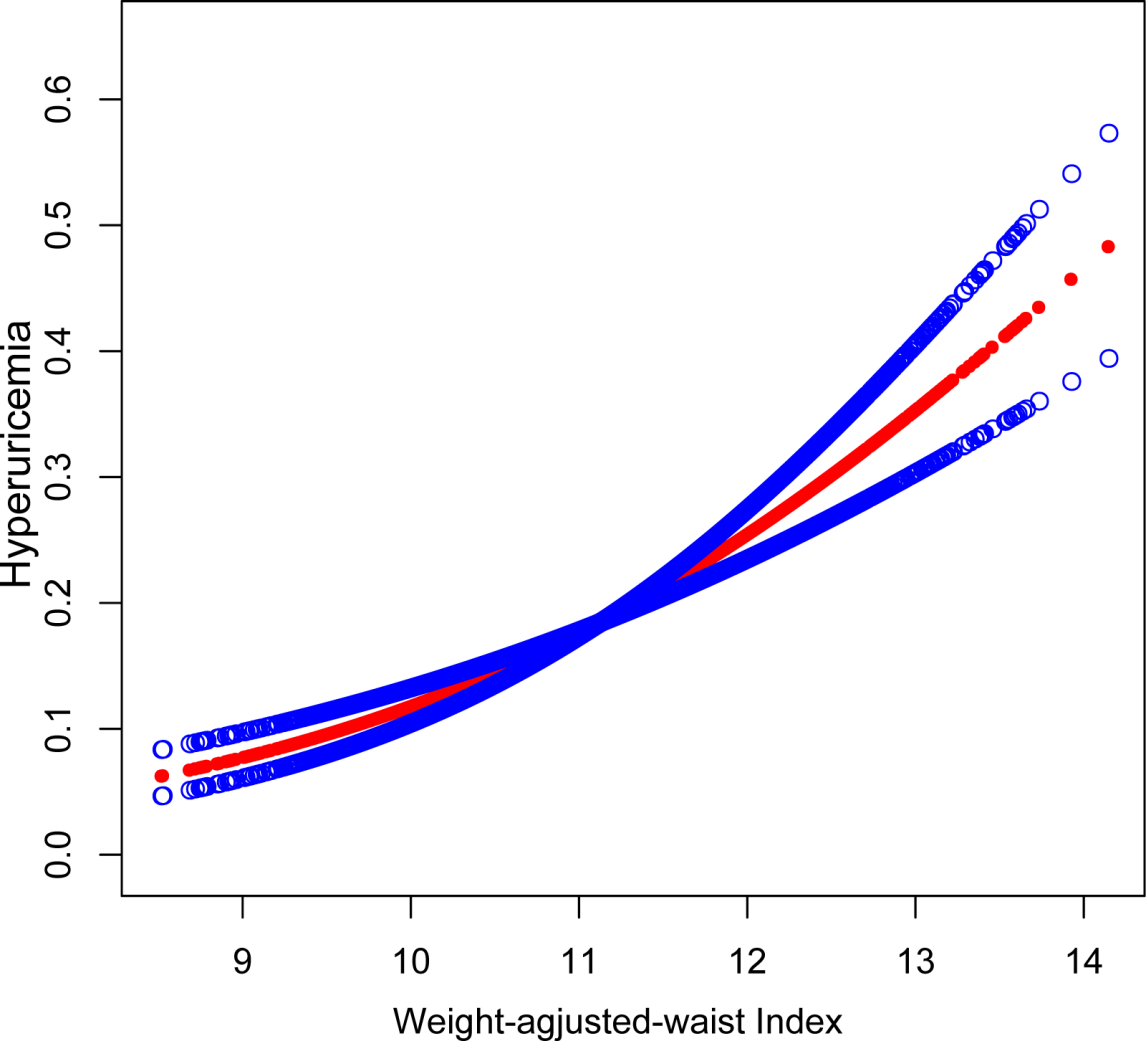
The association between WWI and hyperuricemia. The solid red line represents the smooth curve fit between variables. Blue bands represent the 95% of confidence interval from the fit. Controlled Attenuation Parameter; Weight-adjusted-waist index, WWI.

**Table 2** presents the outcomes of multivariate logistic regression analyses employing three models. A robust positive correlation was established between WWI and the probability of hyperuricemia, with statistical significance maintained across all three models. Following full adjustment, subjects presenting with a unit higher WWI exhibited a 60% increased risk of developing hyperuricemia [OR: 1.60, 95% CI: 1.42-1.80]. When WWI was categorized into tertiles, participants falling into the highest WWI tertile demonstrated a significantly elevated risk, 1.41-fold higher compared to those in the lowest tertile [OR: 2.41, 95% CI: 1.88-3.08] (**Table 2**). Additionally, we employed a generalized model with a smooth curve fitting to corroborate the non-linear relationship between WWI and hyperuricemia. The findings affirmed a non-linear positive correlation between WWI and hyperuricemia (**Figure 2**).

**Table 2:** Association between weight-adjusted-waist index and hyperuricemia.

Exposure	Model 1 [OR (95% CI)]	Model 2 [OR (95% CI)]	Model 3 [OR (95% CI)]
**Continuous WWI**	1.60 (1.49, 1.72)	1.61 (1.48, 1.75)	1.60 (1.42, 1.80)
WWI classification			
**Tertile 1**	Reference	Reference	Reference
**Tertile 2**	1.66 (1.41, 1.95)	1.56 (1.31, 1.84)	1.43 (1.13, 1.81)
**Tertile 3**	2.55 (2.19, 2.98)	2.39 (2.01, 2.85)	2.41 (1.88, 3.08)

Model 1: no covariates were adjusted. Model 2: age, gender, and race were adjusted. Model 3: age, gender, race, education level, income to poverty ratio, smoking behavior, drinking behavior, hypertension, fast glucose, glycohemoglobin, white blood cell, hemoglobin, platelets, serum total bilirubin, serum creatinine, total cholesterol, triglyceride, high-density lipoprotein cholesterol, low-density lipoprotein cholesterol were adjusted.

WWI, weight-adjusted-waist index.

### 3.3 Subgroup analyses

To examine the consistency of the relationship between WWI and hyperuricemia within the overall population and identify potential population-specific parameters, we conducted subgroup analysis and interaction tests stratified by age, sex, hypertension, and drinking behaviour (**Table 3**). The findings indicate an inconsistent association. While the positive correlation between WWI and hyperuricemia remained valid across all subgroups, this relationship was not statistically significant in the subgroup of non-alcohol consumers (P>0.05). Furthermore, no significant interaction was detected between any of the stratified parameters, indicating that the association between WWI and hyperuricemia is independent of age, sex, hypertension, and drinking behaviour (P>0.05).

**Table 3:** Subgroup analysis of the association between weight-adjusted-waist index and hyperuricemia.

Subgroup	OR (95%CI)	*P* for interaction
**Sex**		0.920
Male	1.67 (1.40, 1.99)	
Female	1.65 (1.40, 1.94)	
**Age(years)**		0.178
20-44	1.89 (1.55, 2.31)	
45-59	1.68 (1.34, 2.11)	
≥60	1.46 (1.21, 1.77)	
**Hypertension**		0.115
No	1.69 (1.46, 1.95)	
Yes	1.38 (1.12, 1.69)	
**Drinking behavior**		0.110
No	1.13 (0.73, 1.76)	
Yes	1.65 (1.46, 1.87)	

Age, gender, race, education level, income to poverty ratio, smoking behavior, drinking behavior, hypertension, fast glucose, glycohemoglobin, white blood cell, hemoglobin, platelets, serum total bilirubin, serum creatinine, total cholesterol, triglyceride, high-density lipoprotein cholesterol, low-density lipoprotein cholesterol were adjusted. In the subgroup analyses, the model is not adjusted for the stratification variable itself.

### 3.4 Equations

The equations should be inserted in editable format from the equation editor.


$$\text{WWI}=\frac{\text{WC}}{\sqrt{\text{Weight}}}$$

WC: Waist circumference (cm);Weight (kg)

## 4 Discussion

Our study aimed to investigate the correlation between WWI and hyperuricemia among non-institutionalized civilians in the United States. In our cross-sectional study that involved 7437 individuals, we found that an elevated WWI was significantly associated with an increased likelihood of hyperuricemia. A nonlinear positive relationship was identified between WWI and hyperuricemia, and this relationship remained stable in the fully adjusted model. In the subgroup analyses, all stratification variables, such as age, sex, and hypertension, didn’t affect the consistency of the relationship between WWI and hyperuricemia, and the positive association was consistent across these subgroups. However, this positive association was not observed among non-drinkers, possibly due to their lower purine intake. Our findings provide evidence suggesting that WWI may be a valid predictor of hyperuricemia. Remarkably, this research represents the inaugural cross-sectional study designed specifically to explore the potential association between WWI and hyperuricemia.

The increasing prevalence of hyperuricemia with rising BMI has been documented in several studies (20–22). A cross-sectional survey conducted across nine provinces in China revealed that patients with hyperuricemia had a 2.09-fold greater risk of obesity compared to those with normal uric acid levels (23). Regrettably, BMI despite being a prevalently employed anthropometric parameter, lacks the capability to differentiate between lean tissue and adipose tissue mass. WC has been shown in certain studies to predict the development of hyperuricemia (24, 25). Because WC is often used to estimate visceral fat indirectly, some studies think its prediction of abdominal subcutaneous fat mass can be improved by incorporating BMI into the model (26). Nevertheless, a recent study suggested that the significant association with new-onset hyperuricemia may disappear when WC is included in the model along with BMI (27). Additionally, emerging indicators have also been used to explore the relationship between obesity and hyperuricemia. A 3-year study in women found neck circumference to be an independent risk factor for future hyperuricemia, rather than WC (28). Another Chinese study revealed that the waist-to-height ratio (WHtR) was a better and independent predictor of hyperuricemia than BMI and WC (29). Despite evidence suggesting that these anthropometric measures are correlated with hyperuricemia, the effectiveness of these indicators needs to be confirmed through larger clinical studies and practical application. In recent years, there have been few related studies to demonstrate the potential of WWI as a novel obesity index. A comprehensive cohort study conducted on a national scale in Korea, involving 465,629 participants, ascertained that WWI as the superior predictive indicator for both cardiometabolic disease and mortality rates, outperformed BMI, WC, and WHtR (10). In addition, the result of a 10-year follow-up study also showed that WWI was associated with a higher risk for all-cause mortality, providing further evidence that early medical intervention may improve outcomes in high WWI populations (30). Benefiting from its simple calculation and powerful power to predict disease development, WWI holds great promise as a potential anthropometric indicator.

Hyperuricemia and various metabolic diseases share a mutual relationship in terms of risk factors, with the link between obesity and hyperuricemia gaining significant attention (31–33). Several mechanisms underpin the interplay between obesity and hyperuricemia. In terms of pathological and physiological mechanisms, the imbalance between calorie intake and energy expenditure in obesity leads to excessive accumulation of abdominal and visceral fat. This increases the total nucleic acid metabolism, thereby boosting uric acid synthesis through purine metabolism. Additionally, alterations in glomerular hemodynamics and over-activation of the renin-angiotensin-aldosterone system can prompt obesity-related nephropathy in some individuals. Long-term implications could lead to glomerular atherosclerosis, resulting in reduced renal uric acid excretion (34). At the cellular and molecular level, uric acid, though an antioxidant, can become a pro-oxidant in an obese state. This transformation directly engages in adipocyte proliferation and oxidative stress response, the main contributors to obesity and insulin resistance. Meanwhile, insulin resistance can trigger hyperuricemia by affecting the renal excretion of uric acid (35, 36). Thus, a hyperuricemia-induced oxidative stress response may play a role in the evolution of obesity. Adipokines, hormones secreted by adipocytes, also have a crucial role in energy metabolism and obesity (37). Substantial evidence shows that adipokines, such as adiponectin and soluble leptin receptors, may be associated with changes in uric acid levels (38). Though the specific interaction mechanism between hyperuricemia and obesity remains unclear, it is clinically established that hyperuricemia is closely related to obesity and metabolic syndrome (39). Consequently, the assessment of obesity is necessary to predict hyperuricemia risk, and according to the findings of this study, WWI could serve as a promising obesity indicator.

Our study possesses distinct strengths when compared to previous research in the field. Utilizing a sizable sample from the NHANES dataset, we have captured a representation of the United States population, accounting comprehensively for the sample design and weighting. This provides greater generalizability of our findings to the larger United States demographic. In our analytical approach, we have employed multivariate logistic regression models adjusting for a range of relevant covariates, thus examining the impact of WWI on hyperuricemia. Nonetheless, we acknowledge several constraints inherent in our research. The cross-sectional design restricts us from deducing causal relationships between WWI and hyperuricemia. Further, even with rigorous adjustment for possible covariates, it is important to note that uric acid levels can be modulated by numerous factors, including but not limited to, the use of urate-lowering drugs, the duration of alcohol consumption or smoking cessation, and various socio-environmental variables. We were unable to completely eliminate the influence of these potential confounding variables. Moreover, the overall health and comorbidity profile of participants, not available in the NHANES dataset, might have influenced our results. This lack of data limited our ability to further investigate our hypothesis considering the influence of chronic renal diseases, rheumatic diseases, and other similar conditions. WWI is currently not as prevalently used as BMI and WC in assessing obesity and central obesity in routine clinical practice. Thus, additional clinical studies are warranted to elucidate the advantages and disadvantages of WWI. Lastly, the scope of our findings is fundamentally limited to the United States population, according to the nature of the NHANES database. Therefore, the applicability of our conclusions to diverse ethnic groups or countries outside the United States requires further exploration.

## 5 Conclusion

This study provides compelling evidence that elevated WWI levels are significantly associated with an increased probability of hyperuricemia, thus proposing WWI as a potential biomarker for hyperuricemia in the adult population of the United States. However, these findings necessitate substantiation through future prospective research studies.

## Data availability statement

The original contributions presented in the study are included in the article/supplementary material. Further inquiries can be directed to the corresponding authors.

## Ethics statement

Ethical approval was not required for the study involving humans in accordance with the local legislation and institutional requirements. Written informed consent to participate in this study was not required from the participants or the participants’ legal guardians/next of kin in accordance with the national legislation and the institutional requirements.

## Author contributions

YD contributed to conception, design, data collection, data analysis, data interpretation, and critically revised the manuscript. ZX contributed to conception, design, and drafted the manuscript. XZ contributed to conception and critically revised the manuscript. YLu, RX, and YLi contributedto conception and critically revised the manuscript. All authors contributed to the article and approved the submitted version.

## References

[1] Joosten LAB Crişan TO Bjornstad P Johnson RJ. Asymptomatic hyperuricaemia: A silent activator of the innate immune system. Nat Rev Rheumatol (2020) 16(2):75–86. doi: 10.1038/s41584-019-0334-3 31822862 PMC7075706

[2] Anders HJ Li Q Steiger S. Asymptomatic hyperuricaemia in chronic kidney disease: mechanisms and clinical implications. Clin Kidney J (2023) 16(6):928–38. doi: 10.1093/ckj/sfad006 PMC10229286 37261000

[3] Piani F Agnoletti D Borghi C. Advances in pharmacotherapies for hyperuricemia. Expert Opin Pharmacother (2023) 24(6):737–45. doi: 10.1080/14656566.2023.2197591 36999206

[4] Song J Jin C Shan Z Teng W Li J. Prevalence and risk factors of hyperuricemia and gout: A cross-sectional survey from 31 provinces in mainland China. J Transl Int Med (2022) 10(2):134–45. doi: 10.2478/jtim-2022-0031 PMC9328039 35959454

[5] Heymsfield SB Wadden TA. Mechanisms, pathophysiology, and management of obesity. N Engl J Med (2017) 376(3):254–66. doi: 10.1056/NEJMra1514009 28099824

[6] Ward ZJ Bleich SN Cradock AL Barrett JL Giles CM Flax C. Projected U.S. State-level prevalence of adult obesity and severe obesity. N Engl J Med (2019) 381(25):2440–50. doi: 10.1056/NEJMsa1909301 31851800

[7] De Lorenzo A Gratteri S Gualtieri P Cammarano A Bertucci P Di Renzo L. Why primary obesity is a disease? J Transl Med (2019) 17(1):169. doi: 10.1186/s12967-019-1919-y 31118060 PMC6530037

[8] Wharton S Lau DCW Vallis M Sharma AM Biertho L Campbell-Scherer D. Obesity in adults: A clinical practice guideline. Cmaj (2020) 192(31):E875–e91. doi: 10.1503/cmaj.191707 PMC7828878 32753461

[9] Moltrer M Pala L Cosentino C Mannucci E Rotella CM Cresci B. Body mass index (Bmi), waist circumference (Wc), waist-to-height ratio (Whtr) E waist body mass index (Wbmi): which is better? Endocrine (2022) 76(3):578–83. doi: 10.1007/s12020-022-03030-x 35304685

[10] Park Y Kim NH Kwon TY Kim SG. A novel adiposity index as an integrated predictor of cardiometabolic disease morbidity and mortality. Sci Rep (2018) 8(1):16753. doi: 10.1038/s41598-018-35073-4 30425288 PMC6233180

[11] Qin Z Chang K Yang Q Yu Q Liao R Su B. The association between weight-adjusted-waist index and increased urinary albumin excretion in adults: A population-based study. Front Nutr (2022) 9:941926. doi: 10.3389/fnut.2022.941926 36034904 PMC9412203

[12] Kim NH Park Y Kim NH Kim SG. Weight-adjusted waist index reflects fat and muscle mass in the opposite direction in older adults. Age Ageing (2021) 50(3):780–6. doi: 10.1093/ageing/afaa208 33035293

[13] Campion EW Glynn RJ DeLabry LO. Asymptomatic hyperuricemia. Risks and consequences in the normative aging study. Am J Med (1987) 82(3):421–6. doi: 10.1016/0002-9343(87)90441-4 3826098

[14] Chen JH Pan WH Hsu CC Yeh WT Chuang SY Chen PY. Impact of obesity and hypertriglyceridemia on gout development with or without hyperuricemia: A prospective study. Arthritis Care Res (Hoboken) (2013) 65(1):133–40. doi: 10.1002/acr.21824 22933424

[15] Lin KC Lin HY Chou P. Community based epidemiological study on hyperuricemia and gout in kin-hu, kinmen. J Rheumatol (2000) 27(4):1045–50. 10782835

[16] Tai V Narang RK Gamble G Cadzow M Stamp LK Merriman TR. Do serum urate-associated genetic variants differentially contribute to gout risk according to body mass index? Analysis of the uk biobank. Arthritis Rheumatol (2020) 72(7):1184–91. doi: 10.1002/art.41219 32017447

[17] Zipf G Chiappa M Porter KS Ostchega Y Lewis BG Dostal J. National health and nutrition examination survey: plan and operations, 1999-2010. Vital Health Stat 1 (2013) 56):1–37. 25078429

[18] Bardin T Richette P. Definition of hyperuricemia and gouty conditions. Curr Opin Rheumatol (2014) 26(2):186–91. doi: 10.1097/bor.0000000000000028 24419750

[19] Fryar CD Ostchega Y Hales CM Zhang G Kruszon-Moran D. Hypertension prevalence and control among adults: United States, 2015-2016. NCHS Data Brief (2017) 289):1–8. 29155682

[20] Yang L He Z Gu X Cheng H Li L. Dose-response relationship between bmi and hyperuricemia. Int J Gen Med (2021) 14:8065–71. doi: 10.2147/ijgm.S341622 PMC8594780 34795514

[21] Kuwabara M Kuwabara R Niwa K Hisatome I Smits G Roncal-Jimenez CA. Different risk for hypertension, diabetes, dyslipidemia, and hyperuricemia according to level of body mass index in Japanese and american subjects. Nutrients (2018) 10(8):1011. doi: 10.3390/nu10081011 30081468 PMC6115805

[22] Chen Y Zhang N Sun G Guo X Yu S Yang H. Metabolically healthy obesity also has risk for hyperuricemia among chinese general population: A cross-sectional study. Obes Res Clin Pract (2016) 10 Suppl 1:S84–s95. doi: 10.1016/j.orcp.2016.03.008 27061989

[23] Chen T. Correlation study of uric acid level and metabolic syndrome. Peking Union Med Coll (2009).

[24] Zhou Z Li K Li X Luan R Zhou R. Independent and joint associations of body mass index, waist circumference, waist-height ratio and their changes with risks of hyperuricemia in middle-aged and older chinese individuals: A population-based nationwide cohort study. Nutr Metab (Lond) (2021) 18(1):62. doi: 10.1186/s12986-021-00590-z 34120647 PMC8201932

[25] Wang YY Li L Cui J Yin F Yang F Yuan DM. Associations between anthropometric parameters (Body mass index, waist circumference and waist to hip ratio) and newly diagnosed hyperuricemia in adults in qingdao, China: A cross-sectional study. Asia Pac J Clin Nutr (2020) 29(4):763–70. doi: 10.6133/apjcn.202012_29(4).0011 33377370

[26] Berentzen TL Ängquist L Kotronen A Borra R Yki-Järvinen H Iozzo P. Waist circumference adjusted for body mass index and intra-abdominal fat mass. PloS One (2012) 7(2):e32213. doi: 10.1371/journal.pone.0032213 22384179 PMC3286444

[27] Li Q Li R Zhang S Zhang Y Liu M Song Y. Relation of bmi and waist circumference with the risk of new-onset hyperuricemia in hypertensive patients. Qjm (2022) 115(5):271–8. doi: 10.1093/qjmed/hcaa346 33486528

[28] An P Chen K Wang A Jin X Chen Y Gu W. Neck Circumference Is an Independent Risk Factor for Hyperuricemia within 3 years in Women: A Longitudinal Study. Clin Rheumatol (2020) 39(12):3757–67. doi: 10.1007/s10067-020-05095-3 32458241

[29] Huang ZP Huang BX Zhang H Zhu MF Zhu HL. Waist-to-height ratio is a better predictor of hyperuricemia than body mass index and waist circumference in chinese. Ann Nutr Metab (2019) 75(3):187–94. doi: 10.1159/000504282 31743929

[30] Cai S Zhou L Zhang Y Cheng B Zhang A Sun J. Association of the weight-adjusted-waist index with risk of all-cause mortality: A 10-year follow-up study. Front Nutr (2022) 9:894686. doi: 10.3389/fnut.2022.894686 35694172 PMC9174751

[31] Dong H Xu Y Zhang X Tian S. Visceral adiposity index is strongly associated with hyperuricemia independently of metabolic health and obesity phenotypes. Sci Rep (2017) 7(1):8822. doi: 10.1038/s41598-017-09455-z 28821853 PMC5562916

[32] Lee JH. Prevalence of hyperuricemia and its association with metabolic syndrome and cardiometabolic risk factors in korean children and adolescents: analysis based on the 2016-2017 korea national health and nutrition examination survey. Korean J Pediatr (2019) 62(8):317–23. doi: 10.3345/kjp.2019.00444 PMC6702117 31319650

[33] Duan Y Liang W Zhu L Zhang T Wang L Nie Z. Association between serum uric acid levels and obesity among university students (China). Nutr Hosp (2015) 31(6):2407–11. doi: 10.3305/nh.2015.31.6.8734 26040345

[34] Martínez-Montoro JI Morales E Cornejo-Pareja I Tinahones FJ Fernández-García JC. Obesity-related glomerulopathy: current approaches and future perspectives. Obes Rev (2022) 23(7):e13450. doi: 10.1111/obr.13450 35362662 PMC9286698

[35] Johnson RJ Nakagawa T Sanchez-Lozada LG Shafiu M Sundaram S Le M. Sugar, uric acid, and the etiology of diabetes and obesity. Diabetes (2013) 62(10):3307–15. doi: 10.2337/db12-1814 PMC3781481 24065788

[36] Gong M Wen S Nguyen T Wang C Jin J Zhou L. Converging relationships of obesity and hyperuricemia with special reference to metabolic disorders and plausible therapeutic implications. Diabetes Metab Syndr Obes (2020) 13:943–62. doi: 10.2147/dmso.S232377 PMC7125338 32280253

[37] Son DH Ha HS Park HM Kim HY Lee YJ. New markers in metabolic syndrome. Adv Clin Chem (2022) 110:37–71. doi: 10.1016/bs.acc.2022.06.002 36210076

[38] Cong R Zhang X Song Z Chen S Liu G Liu Y. Assessing the causal effects of adipokines on uric acid and gout: A two-sample mendelian randomization study. Nutrients (2022) 14(5):1091. doi: 10.3390/nu14051091 35268067 PMC8912555

[39] Fahed G Aoun L Bou Zerdan M Allam S Bou Zerdan M Bouferraa Y. Metabolic syndrome: updates on pathophysiology and management in 2021. Int J Mol Sci (2022) 23(2):786. doi: 10.3390/ijms23020786 35054972 PMC8775991

